# The evaluation of surface sealants’ effect on the surface roughness of Nano-hybrid composite after polishing with One-Step system (*in-vitro*)

**DOI:** 10.4317/jced.54858

**Published:** 2018-07-01

**Authors:** Kaveh Khalaj, Mahsima Tayefi-Nasrabadi, Armin Soudi

**Affiliations:** 1 Assistant Professor of Operative Dentistry, School of Dentistry, Tehran University of Medical Sciences, Tehran, Iran; 2 DDS, Department of Oral and Maxillofacial Radiology, School of Dentistry, Shahid Beheshti University of Medical Sciences; 3 DDS , Department of Orthodontics, School of Dentistry, Tehran University of Medical Sciences, Tehran, Iran

## Abstract

**Background:**

The clinical use of composite resins has increased substantially due to increased esthetic demands by patients, improvements in formulation, and simplification of bonding procedures. Use of surface sealants is recommended to improve surface smoothness of composite restorations. The aim of this study was to evaluate the effect of surface sealants on the surface roughness of nano-hybrid composite after polishing with One-Step system.

**Material and Methods:**

Using a silicon mold, 56 specimens of 10 mm diameter and 2 mm height were prepared from Grandio nano-hybrid composite (Voco, Cuxhaven, Germany) with A2 shade. Specimens were randomly divided in 2 groups. The surface of specimens were polished with VOCO One-step system. One group of specimens assigned as control group and received no more surface treatment. Surface sealant PermaSeal (Ultradent, USA) was applied on the surface of specimens of the other group as experimental group. Specimens were stored in Ringer’s solution at 37°C for 24 hours. The specimens were subjected to artificial accelerated aging with thermocycling method (3000 cycles, between 5 and 55°C) and then 100000 cycles of tooth brushing. A Profilometer was used to measuring the surface roughness of specimens before and after aging procedures.

**Results:**

The surface roughness changes of control group were significant before and after aging (*P*<0.05). The mean final surface roughness of control group was unacceptable clinically while it was acceptable in experimental group.

**Conclusions:**

The use of surface sealants on nano-hybrid composite causes noticeable reduces in surface roughness of composite.

** Key words:**Surface-penetrating sealant, surface roughness, nano-hybrid, composites, one-step polishing.

## Introduction

The advent of acid-etch technique (Buonocore, 1955), followed by the introduction of Bis-GMA composite resins (Bowen, 1956), was undoubtedly a major step in adhesive dentistry (1). As a result of increasing aesthetic requirements of patients and evolution of physical, optical and mechanical properties of composite resins, these materials have become known as one of the most used materials in dentistry (2). Extensive use of composite resins is due to their mercury-free structure, electrical non-conductivity and resistance to corrosion (2,3). These resin materials have evolved from macro-filled to micro-filled and from hybrids to micro-hybrids, and new materials such as packable and nano-filled composites have entered the market (3). Manufacturers have reduced the mean size of inorganic particles in order to reduce surface roughness to achieve better optical properties and decrease surface abrasion of composites (2,4). Recent development in composite resins is associated with Nanotechnology, and has led to the production of materials that have better mechanical strength and longer surface durability with higher polish (4).

In clinical conditions, lifetime of restorations is typically associated with acceptable polishing properties that provide a smooth surface (5). Finishing and polishing procedures are necessary to achieve the beauty and durability of tooth-colored restorations (6). Color stability is critical for success in any aesthetic restoration; in fact, color change is the main reason for the replacement of anterior restorations (7). The marginal finishing, surface roughness and surface integrity, as much as the physicochemical properties of the material itself, can affect the plaque retention. This, in turn, plays a significant role in the development of periodontal diseases and recurrent dental caries (2,3). Greater values of surface roughness (> 0.2 μm) has been reported as a risk factor for high levels of plaque accumulation on restorative materials, as the main factor in color changing of resin restorations (8,9). Therefore, maintaining the smooth surface of a restoration is the most important factor in its success. Composites are polished to create a functional occlusal relationship and to establish a physiological contour in balance with supporting tissues. In addition, suitable and high-gloss counter gives a normal appearance similar to the natural tissue of the tooth (3). Since the introduction of bonding systems and resin composites, abrasion and micro leakage have been the main initial clinical limitations, especially in posterior restorations. The restoration roughness and surface absorption of oral fluids can also allow colorant factors to penetrate into the material (4).

To overcome this problem, the use of a thin layer of resin, with a low viscosity and high moisturizing ability, has been recommended for polymerizing restorations, including Bis-GMA, UDMA and TEG-DMA without filler particles. Surface-penetrating sealant or rebound agent should be able to fill the structural micro defects and micro fissures formed during the insertion, finishing and polishing techniques with the capillary effect. This approach is thought to improve surface smoothness by providing a more uniform and organized surface (1,5,10). Surface penetration sealants have been developed to avoid or minimize the abrasion rate of resin composites by filling the micro-defects on the surface of the restoration and reduction of micro leakage at the tooth-restoration contact surface. The goal is not to close open margins; but to fill the micro irregularities that may be present in the cavo-surface margins, which are formed due to correction and polishing. On the other hand, by creating a layer on the restoration surface, Surface sealant protects the surface and increases its longevity. It can also be re-used in patients’ recall sessions to maintain restoration integrity (11). It is said that the sealant substance is polymerized without creating an oxygen inhibitor layer and shows a conversion rate of 80% (12). In addition, some surface sealants act as a chemical gloss to reduce surface roughness (4).

Following the insertion of direct and indirect aesthetic restorations, often occlusive and marginal correction of the restoration surface is required. Rotary and rubber polishing abrasive tools are used to create a surface glow that has been lost during the correction. Recently, one-step polishing systems containing diamond particles and synthetic silicon rubbers have been introduced to the market, which are able to provide hybrid composites the glow of microfill composites and reduce the clinical steps and time spent on polishing. The manufacturers have called this system “one-step polishing system” because the high luminosity, contour and finishing and polishing procedures are possible using a single instrument. These type of polishing systems make it possible to create a smooth surface in the least time and using only one tool (13).

Since the introduction of resin composites, many studies have been conducted to achieve a smooth surface on the restoration and have yielded various, sometimes contradictory, results. Therefore, determining whether the use of surface sealant offers better results for aesthetic restorations remains questionable. The aim of this study is to determine the effect of surface sealant on surface roughness of a nano-hybrid composite after polishing with a one-step system under aging processes in the *in-vitro* environment.

## Material and Methods

-Experimental Design

The samples under study were two groups.

1. One-step polishing, 

2. Applied surface sealant after One-step polishing, consisted of 56 specimens (n = 28), made in random sequence. The quantitative response variable was composite surface roughness evaluated by means of a proﬁlometer before and after the thermocycling and tooth brushing abrasion test.

-Materials

The materials used in this study are outlined in  Table 1.

**Table 1:**
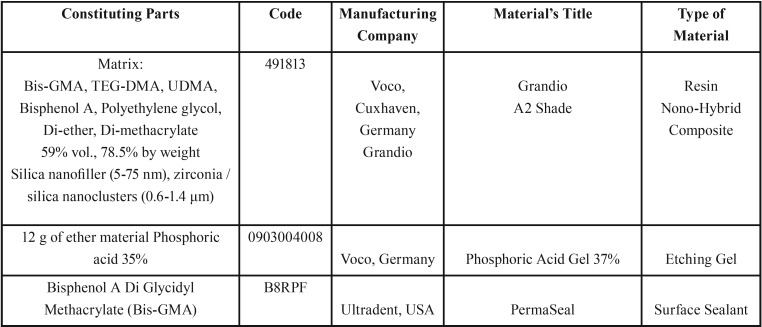
Materials used in this study and their characteristics.

-Specimen preparation

Using a medical grade silicone mold placed on a glass plate and a polyester matrix strip (in order to support the mold), 56 round composite disks (Voco, Cuxhaven, Germany) with A2 Shade were made, measuring 10 mm in diameter and 2 mm in height. The composite was deposited in a single layer inside the mold with the help of Optra Sculp (Ivoclar-vivadent) instrument. To ensure a smooth surface in the samples, before light curing, the upper surface of molds were covered with a second transparent matrix and another glass slab was placed on it, and to remove the excess composite and to create a standard smooth surface, a weight of one Kilogram was put on the collection for 30 seconds. Afterward, the weight and the top glass slab were removed and the samples were cured for 40 seconds through the Mylar strip by LED light cure (Woodpecker) with the intensity of 800 mw / cm2 according to the instruction of factory. The tip of the light cure device was placed at a distance of less than 5 mm perpendicular to the composite surface, and the intensity of the output light of the device was monitored by a radiometer initially and between each of the 5 samples. The surface of the samples was polished by a Lens disk of one-step polishing system of Dimanto (Voco, Cuxhaven, Germany), using a low-speed hand piece without cooler for 20 seconds according to the instruction of factory. At first, the average pressure was applied, and in the final stage of polishing, the imposed pressure dropped to achieve the gloss. All specimen preparation and finishing-polishing procedures were carried out by the same trained person to avoid interpersonal differences in performance. Then the samples were completely washed and dried by air pressure.

The samples were randomly divided into two groups, each containing 28 specimens and at this stage a group of samples were placed in 37ºC Ringer’s solution as a control group. The other group (experimental group) was etched by phosphoric acid 37% (Voco, Germany) for 20 seconds according to the instruction of the factory, then washed with water syringe for 20 seconds, and dried slowly with absorbent paper and air syringe. A thin layer of PermaSeal surface sealant (Ultradent, USA) was placed on the surface of the samples using a Black Micro FX (Ultradent, USA) tip, with rubbing motions for 5 seconds, and was thinned with gentle air pressure and cured for 20 seconds by the LED light device according to the instruction of the factory. Then, the samples were removed from the molds and the lateral flushes of the composite and the surface sealant were slowly removed using the Diamond Fissure Bur 008.

All samples were kept in Ringer’s solution for 24 hours in a Kavoosh MEGA incubator (kavoosh industry,Tehran,Iran) at 37 ± 1°C.

After leaving the incubator, the samples were completely washed with water pressure and dried by absorbent paper.

-Baseline roughness (R) measurements

Each of the samples was mounted using a special mold in the center of a 3 x 4 cm acrylic cast, so that 1 mm inside the acrylic dipped. The samples were washed with water pressure and dried with air pressure. Then, surface roughness of the samples was measured using a TR200 Time Profilometer (CV Instruments Europe BV, Indonesia) in an area of 0.8 mm x 1 mm. For each sample, the Ra parameter was repeated in three areas of the disc surface, and the obtained mean values were considered as the initial surface roughness of the sample.

-Artificial aging

Artificial aging of the samples was done using the TC-300 Thermo-Cycle (Vafaei industrial, Iran) device. Samples were exposed to intermittent thermal tensions in thermocycle device in Ringer’s 5 and 55°C solution at 3000 cycles (with a transfer time of 10 seconds between two baths and 30 seconds in each bath). After completing the thermocycle stages, all specimens were simulated under the brushing procedure by an electric toothbrush (Oral-B, cross action with round end, medium-bristle-tip) with a frequency of 50-60 Hz, equal to 300-360 bpm in a chamber containing distilled water and Colgate toothpaste with 2: 1 ratio. A hand made holder was used to hold the toothbrush tip so that bristles of the toothbrush were perpendicular to the surface of the specimen. The toothbrush tip was placed in order to create uniform abrasion on the surface of the samples. Every 10,000 cycles were considered equivalent to one year of brushing. After applying 100,000 cycles continuously under a force of 200 g (force applied by placing a 200 g weight on the toothbrush tip), the samples were completely washed off with water pressure and dried before measuring the surface roughness by the air syringe. According to Heintze *et al.*, a significant change in surface composite tension occurs after 72,000 cycles. (14)

-Final roughness measurements

Finally, the surface roughness of the samples was again measured by the Profilometer device. Ra values were measured in three regions of the surface of each disk and the mean values were considered as the final surface roughness of the samples.

-Statistical analysis

Data were analyzed by SPSS software version 20. After descriptive statistics, t-test, chi-square test was used. *P* value less than 0.05 was considered as statistically significant difference.

## Results

The study samples consist of two groups of 28 round composite disks. Samples of the experimental group entered the study for examining the surface roughness after one-step polishing and applying the surface sealant during artificial aging processes and the control group samples were included for studying surface roughness after a one-step polishing during the artificial aging process.

For the experimental group, paired t-test did not show remarkable difference between surface roughness before and after aging processes (*P*=0.065). The value of *P*=0.025 was obtained using the independent t-test for the changes in surface roughness from baseline to the final stage for the control group. Regarding the mean values, we conclude that the amount of surface roughness changes through the artificial aging processes is greater in the control group ( Table 2).

**Table 2:**
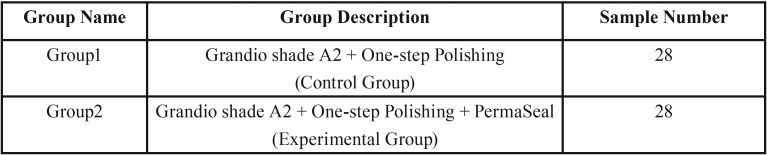
Comparison of baseline (initial) and final (after aging processes) Surface roughness in control and Experimental groups.

At the baseline, using the independent t-test, the value of *p* = 0.005 was obtained. The value of p indicates a significant difference in the baseline surface roughness (before the aging process) between two groups. With regard to the mean values, we conclude that the surface roughness before the aging process is greater in the control group. The independent t-test showed a significant difference in the final surface roughness (after the aging process) between two groups (*P*= 0.00) ( Table 3).

**Table 3:**
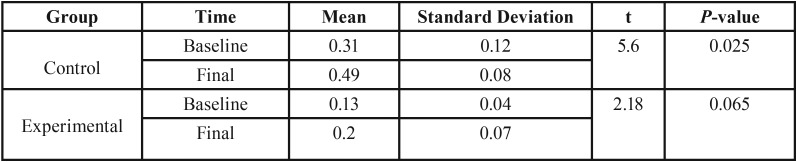
Comparison of surface roughness in the control and experimental groups before and after aging processes.

 Table 4 shows the comparison of surface roughness changes (ΔRa) before and after the aging process in the control and experimental groups. As it is indicated, mean changes in surface roughness before and after aging is significant (*p*=0.025) ( Table 5).

**Table 4:**
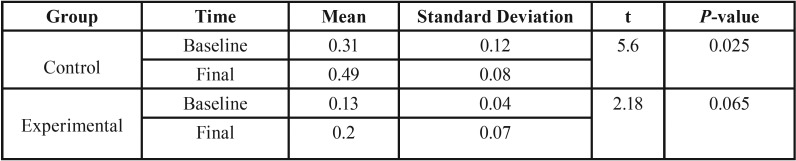
Comparison of surface roughness changes (ΔRa) before and after the aging process in the control and experimental groups.

**Table 5:**
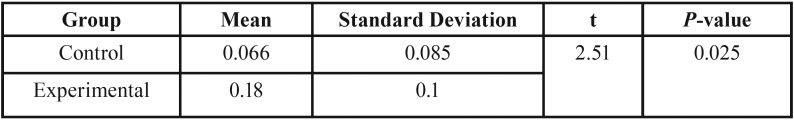
Comparison of Surface Roughness changes (ΔRa) before and after the Aging Process In the control and experimental groups.

## Discussion

Composites are widely used in dental treatments due to their aesthetic and adhesion considerations. Following the application of these materials, creation of the smoothest surface is necessary to minimize coloration and plaque accumulation. In addition, suitable counter and a great deal of luster create the appearance of a natural tooth and is a favorite of the patient. It is therefore important to determine which finishing and polishing system creates the best results to maintain the beautiful appearance of the restoration. The use of surface sealant for composites has been proposed to reduce the potential of the coloration and abrasion of composites (15). The use of surface sealant on the surface of composite resin results in a significant enhancement of its surface texture, which is observed by electron microscopy and its thickness is within the range 0 to 70 micrometers (16).

Theoretically, the surface sealant of the composite reinforces the marginal integrity of composite resin restoration. Judes *et al.*, showed that the application of low-viscosity resin on the restoration margins penetrates deeply into micro gapes and surface micro defects. In order for the composite surface sealant to function effectively, it should have suitable wetting, small contact angle and potential for flowing to the minor restoration defects. Finishing and polishing methods produce heat and reduce adhesion resistance against micro-leakage. On the other hand, micro cracks or bubbles may be present in the composite sub-surface layer. Application of the composite surface sealant seals these micro structural defects and prevents composite abrasion (17).

Since the introduction of surface sealants into the market, various laboratory and clinical studies have evaluated these substances under different protocols. These studies seem to agree on the effect of surface sealants on micro-leakage reduction, despite their inability to completely eliminate it (4). The most common way to examine the effects of surface coating on surface texture of the materials, is using the sealants after the polishing process, which provides the ability to achieve surfaces with lower roughness or without any surface defects (5). A rough surface can reduce resistance to abrasion and significantly increase the areas that are prone to plaque accumulation on the restoration surface, and as a result, increases the incidence of oral diseases, apart from making the restoration prone to coloring and loss of lustre (1). Although all polishing systems have advantages and disadvantages, their function varies according to the smooth surface they create.

The results of this study showed that artificial aging processes significantly increase the surface roughness of the composite. This result was consistent with the result of the study by Takeuchi *et al.*, (18). Catelan *et al.*, announced that their artificial aging processes had no effect on surface roughness of the substances of the study. They used UV rays and immersion samples in the coca, orange juice and red wine solutions for artificial aging of the samples (19). While in our study thermal changes and abrasion by toothbrush were used, that these methods are more reliable for simulating the abrasion and thermal conditions of composite in the oral environment, which affects surface roughness.

The data obtained from this article showed that the initial surface roughness of the experimental group (sealed samples) was significantly lower than that of the control group (*P* = 0.005). In the experimental group, the surface roughness of the samples increased after artificial aging processes, but this amount of increase was not statistically significant (*P* = 0.065). In other words, the application of surface sealant retained the smooth surface of the samples during artificial aging processes. However, the surface roughness of the control group samples increased significantly after artificial aging processes (*P* = 0.25). In comparison of the final surface roughness of the two groups, the surface roughness of the samples of control group was higher than that of the experimental group and this difference was statistically significant (*P*= 0.00).

Also, the obtained results were consistent with the results of studies by Attar et al., in 2007. They concluded that the use of surface sealants after polishing the samples reduced the surface roughness of all the studied samples (3). Also, the results of studies by Cilli *et al.*, in 2009 showed that at each step of brushing, the sealed group showed less surface roughness than the unsealed group (20). Also, Perez *et al.*, in 2009 concluded that surface sealant application improved the surface roughness of all examined composites. Perez *et al.*, announced that surface sealant is a valuable tool in creating a polished surface and reducing surface roughness (21). In our study, a nano-hybrid composite and one-step polishing system were used. A one-step polishing system is able to reduce the clinical time spent and create high luster in Nano-composites. Also, our samples underwent 3000 cycles of thermocycling between the 5° and 55° Ringer’s solutions, which, in addition to the abrasive tests, simulated the thermal changes imposed on the composite in the oral environment.

Lopes *et al.*, reported that in toothbrush simulation every 10,000 cycles would be equivalent to one year of brushing (4). Surface roughness of dental substances changes with brushing time and imposed force. Heintz *et al.*, report that 72,000 cycles of brushing are required to make surface changes in the composite (22). In our study, 100,000 brushing cycles equivalent to ten years of brushing in the mouth environment were used. In our study, we tried to simulate the environmental conditions of the composite over time in the mouth environment. Thus, in addition to simulating the abrasion by toothbrush, simulation of thermal changes was also used.

In 2012, Lopez *et al.*, examined the effect of three types of sealants on surface roughness of a Nano-filler composite under brushing procedures. The data from their study showed that there was no significant difference between the control group and the experimental group. They concluded that the surface sealant does not improve surface roughness of Nano-filler composite (23). These results contradicted the results of our research. In their study, the samples were placed in an ultrasonic machine for 10 minutes after doing the artificial aging procedures. While the purpose of our study was to simulate the conditions present in the mouth environment and thus the samples were completely washed with water pressure. Also, in this study, 3000 thermocycling cycles were used because of simulating the thermal changes of the mouth environment. In their study, measurements of surface roughness were done using a SEM device, which is a qualitative measure, while in our study, the quantitative data obtained from the profileometer device were used.

The results of this research were inconsistent with the results of the study by Bagis *et al.* They concluded that the using surface sealants may have no benefit in improving surface roughness and color stability, and the sealed samples may have a higher surface roughness (5). The sealant used in their study contains filler particles of 35 up to 40 micrometers. Also in their study, UV ray was used for aging the samples. While the sealant used in our study did not contain filler particles and this could be a reason for lower roughness of sealed surfaces in our study. Also, in order to simulate the existing conditions in the mouth environment, in our study, abrasion and thermal changes were used for aging the samples.

## Conclusions

This study showed that the surface roughness of the samples sealed by surface sealants before and after artificial aging processes was lower than unsealed samples, and this difference was statistically significant. These findings could confirm the theory that application of surface sealant improves surface roughness of the Nano-hybrid composites.
